# Understanding diverse subjectivities of medical students in seeking composite role models: A Q-methodology study

**DOI:** 10.1371/journal.pone.0339766

**Published:** 2026-02-27

**Authors:** Reihaneh Hasani, Elaheh Mohammadi, Ali Norouzi, Mahboobeh Khabaz Mafinejad

**Affiliations:** 1 Department of Medical Education, School of Medicine, Tehran University of Medical Sciences, Tehran, Iran; 2 Department of Medical Education, School of Medicine, Tehran University of Medical Sciences, Tehran, Iran; 3 Health Professions Education Research Center, Education Development Center, Tehran University of Medical Sciences, Tehran, Iran; 4 Medical Education Development Center (MEDC), Al-Subtain University, International branch of Tehran university of Medical Sciences, Karbala, Iraq,; 5 Social Determinants of Health Research Center, Zanjan University of Medical Sciences, Zanjan, Iran; King Saud bin Abdulaziz University for Health Sciences, SAUDI ARABIA

## Abstract

**Background:**

Role modeling, an integral part of medical education, is a complex and multifaceted process. To unravel the hidden dimensions of role model selection, a comprehensive understanding of students’ subjectivities is crucial. This research aimed to elucidate the dominant subjectivities of medical students when selecting composite role models.

**Methods:**

This study employed the Q-methodology to investigate individuals’ subjectivities and portray them. Initially, we formulated Q-statements based on semi-structured interviews with students and a literature review. In the second step, 31 undergraduate medical students were selected through purposive and snowball sampling. In the next step, data were collected using a questionnaire and a Q-grid. In the fourth step, we categorized individuals using KADE software and performed factor analysis. Finally, the research team utilized factor arrays and combined quantitative and qualitative data to interpret the dominant subjectivities in the selection of composite role models.

**Results:**

This study identifies four dominant subjectivities among 31 undergraduate medical students, explaining 50% of total variance, including: 1) Relying on professional and skillful role models (n = 13, 34% variance): students emphasize professional behavior, responsibility, and strong teaching competence, 2) Relying on popular and famous role models (n = 4, 6% variance): students value fame at micro to macro levels, including clinical excellence and international engagement, 3) Relying on venerable and value-oriented role models (n = 6, 6% variance): students prioritize moral principles, ethical conduct, and alignment with personal values, 4) Relying on social and responsive role models (n = 5, 4% variance): students highlight social engagement, altruism, and a positive impact on society.

**Discussion:**

Our research indicates that multiple factors significantly influence students’ selection of composite role models, with varying subjectivities across four domains. Teachers can use these subjectivities to reflect on the factors involved in role modeling. Medical schools can leverage these identified subjectivities to enhance role modeling and improve teaching and learning.

## Introduction

Role modeling, as a crucial and integral part of medical education [1], impacts various aspects, including improving learning, changing professional attitudes and behaviors, forming professional identity, and providing career orientation [2–6]. As Perry emphasizes, role models are not just catalysts in the teaching process but also architects of professional identity [7]. They instill professional values, which in turn shape the future performance of graduates [8,9]. Their influence is instrumental in forming the professional personality of learners [10]. These impacts, as shown by previous studies, effectively facilitate the transformation of students into doctors [6].

Role modeling has been investigated in studies based on two theories of social learning and cognitive construction. Based on social learning theory, teaching with the help of a role model is rooted in the theories of socialization and observational learning [11]. According to this view, role modeling primarily relies on imitating and observing individuals’ performances, a fundamental concept of Bandura’s social learning theory [12]. According to this theory, individuals can imitate and receive vicarious reinforcement [12]. They can watch others’ behaviors without personal involvement, understand the outcomes, and determine which behaviors to adopt or avoid by observing vicarious reinforcement and punishment [13]. Based on this theory, research in the field of role modeling has traditionally focused on repeated encounters and relationships with individuals who possess specific characteristics for modeling [14–16]. From the perspective of cognitive construction, role modeling is considered a cognitive process in which individuals actively observe, adapt to, and reject the attributes of multiple role models [17]. In this context, the concept of a “composite role model” refers to a cognitively constructed figure that emerges from the integration of multiple factors—such as personal traits, social dynamics, economic conditions, institutional roles, and broader structural influences. Rather than emulating a single individual, learners form a mosaic-like role model shaped by diverse experiences and contextual realities [18]. Evidence shows that, unlike the role model strategy common in studies that seek a “whole” role model, this approach attempts to actively select role models’ desirable attributes and define them in the form of a “composite role model,” and gain insight into what students like are becoming [18,19].

Evidence has shown that role models have been primarily assessed as part of the socialization process in medical education; however, research focusing specifically on role models through the cognitive construction lens is rare [17,20]. Furthermore, many studies have employed descriptive methodology to identify the attributes of an effective role model [21–25]. But since role modeling is a complex and multidimensional process that involves conscious and subconscious elements [26], a comprehensive examination of students’ subjectivities is necessary to understand the hidden dimensions in defining a “composite role model.” To the best of the authors’ knowledge, the selection of role models has not been categorized from the point of view of different spectrums of participants in most studies, and the subjectivities in the definition of a composite role model, the extent of each subjectivity, and the effective and ineffective factors related to individuals’ subjectivity, as examined through Q-methodology, have not yet been explored.

Q-methodology is based on subjectivity, which refers to the communicable and self-referenced viewpoints of individuals, shaped by their personal beliefs, values, opinions, emotions, and preferences. In this approach, each person’s subjectivity emerges from the unique weighting and prioritization they assign to a shared set of statements, allowing researchers to identify distinct patterns of thought across participants [27–29]. Given that the Q-methodology is a suitable method for discovering and exploring individual subjectivities, and for categorizing these factors in comparison with the subjectivities of different participant groups, this research aimed to explain the dominant subjectivities of medical students in selecting role models.

## Methods and materials

### Setting

This study was conducted at the Faculty of Medicine, Tehran University of Medical Sciences (TUMS), Iran. The undergraduate medical education at TUMS is a 7-year competency-based education curriculum divided into basic sciences, pathophysiology, clerkship, and internship. Students spend two years learning theoretical basic sciences and one year studying pathophysiology. After completing the pre-clinical phase, students enter the clinical phase, participating in multiple clinical rotations in major and minor fields at affiliated teaching hospitals and educational health centers. This study was conducted during the clinical phase of undergraduate medical education.

### Study design

This study employed Q-methodology to identify dominant subjectivities among medical students in their selection of composite role models, revealing influential factors behind their subjectivities and choices through weighted Q-statement sorting, in line with the cognitive construction perspective. Q-methodology is particularly suitable for studying and illustrating individuals’ subjectivities [29,30] as it enables researchers to gain a deep and nuanced understanding of participants’ viewpoints on the study topic [31]. Participants rank a set of Q-statements representing diverse aspects of the subject under study, thereby expressing their subjectivities through the Q-sorting process. In this method, individuals categorize statements based on their perceptions, enabling the identification of both shared and divergent viewpoints [30,32]. Medical education research has been done using Q-methodology, including examining the attitude of medical students toward active learning in small groups [33]. The career orientations of medical students [32]. The attitude of medical students toward communication with patients [34]. Patterns of self-regulated learning behaviors [35]. Assessment of residents’ motivation towards teaching [36]. Assessment of tendencies to curriculum changes [37]. The study followed five steps outlined by Watts and Stenner (2012) [38]: Q-set development, participant recruitment, data collection, data analysis, and factor interpretation.

### Step 1: Q-set development

The initial step in Q-methodology involves creating a Q-set, comprising statements about the topic under investigation. This process was conducted in four stages: (1) extracting factors influencing role model selection through interviews, (2) extracting factors through a literature review, (3) developing Q-statements, and (4) revising and finalizing the Q-set.

1-1. *Extracting factors via Interviews:* To identify factors influencing the selection of composite role models, 15 semi-structured interviews were conducted with clinical-phase medical students at TUMS using purposive sampling with a maximum variation strategy to include diverse perspectives (e.g., internship and clerkship phases, genders, and native/non-native status). Inclusion criteria were: (1) being an undergraduate medical student at TUMS, (2) having completed at least three months in the clerkship or internship phase, and (3) willingness to participate. Interviews were scheduled at participants’ convenience (in-person or virtual), preceded by an interview guide and informed consent. With permission, interviews were recorded and lasted 45-60 minutes each, conducted by RH. Sampling continued until data saturation was reached. In this study, to ensure data sufficiency and reach theoretical saturation during the interview process, we adopted several strategies commonly used in qualitative research. First, we did not predetermine a fixed sample size. Instead, we kept conducting interviews until no new codes or themes emerged in the final three consecutive interviews. Second, as preliminary coding and initial data analysis progressed, we routinely asked probing and follow-up questions whenever a topic appeared underexplored or required deeper clarification [39]. This iterative back-and-forth continued until no additional concepts, categories, or properties surfaced in the data. Third, we performed data analysis and coding of the interview transcripts concurrently with the ongoing interviews. This parallel process allowed us to continuously monitor the emergence of new information and verify, on an ongoing basis, whether we had truly achieved data sufficiency and saturation. Transcripts were analyzed line-by-line to extract codes. Two researchers (MKM, RH) independently reviewed codes over two three-hour sessions; disagreements were resolved with a third researcher (EM). Trustworthiness was ensured using Guba and Lincoln’s criteria [40].
1-2. *Extracting Factors via Literature Review:* A search was performed using keywords such as (“Role Model*”) OR (“Role-Model*”) AND (“Medical Education” OR “Medical Student*”) AND (“Select*” OR “Choose” OR “Prefer” OR “Elect”) AND (“Characteristic*” OR “Trait” OR “Attribute”) in PubMed, Scopus, and Google Scholar. Exclusion criteria included: (1) studies on basic principles of role modeling, (2) those examining the impact or importance of role modeling, and (3) role modeling in non-medical fields.Data analysis was carried out using directed content analysis.
1-3. *Developing Q-statements:* Based on the findings of the interviews and literature review, 69 initial Q-statements were written by two researchers (RH, MKM) during three 2-hour sessions. At this stage, to write Q-statements, several criteria are included 1) the statements represent the subject under study, 2) the use of familiar and understandable expressions, 3) the use of unambiguous, simple, and as short as possible expressions, and 4) the use of expressions based on participants’ experiences.
1-4. *Revising and finalizing the Q-set:* involved multiple feedback rounds. First, five medical education experts reviewed statements for relevance, overlap, and completeness in a two-hour meeting. Second, four medical students assessed clarity by reading statements aloud, thinking aloud, and verbal probing; RH noted ambiguities. Finally, in a two-hour research team meeting, statements were revised based on feedback, resulting in 55 finalized Q-statements (aligned with evidence suggesting 20-60 statements) [41]. The final Q-set is shown in S1 Appendix

### Step 2: Participant recruitment

In Q-methodology, participants should be selected in a way that represents the breadth of opinions about the subject under study. The number of participants should be fewer than the number of Q-set statements to ensure robust factor analysis, typically with a ratio of two to five statements per participant [42,43]. Additionally, according to the study by Webler et al., the average number of participants in this method ranges from 12 to 40 [41]. In the current study, 31 students of the undergraduate medical curriculum were selected based on the criteria of 1) being medical students at TUMS, 2) spending at least three months of the internship or internship phase, and 3) being willing to participate in the study using the purposive sampling method. The sample size of 31, which was less than the 55 statements and approximated a ratio of 1.8 statements per participant, was sufficient to identify distinct subjectivities, aligning with the standards of Q-methodology. Purposive sampling was employed, and the snowball method was used to identify additional qualified students for participation in the study.

### Step 3: Data collection

**Fig 1:**
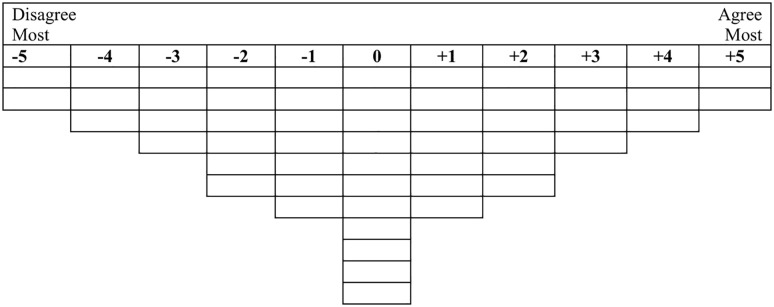
Grid showing the prearranged normal distribution for the Q-sorting.

The data of this study were collected from October to November 2023. The questionnaire included demographic information and a Q-grid using a Likert scale ranging from −5 to +5. Furthermore, the informed consent form was obtained in written form and signed by participants. We assigned a specific number to all the Q-statements developed in the previous stage. Additionally, the Q-set was prepared on an A3 page and provided to the participants, allowing them to compare and sort the Q-statements within the Q-grid easily. Moreover, instructions on how to complete the Q-grid were prepared, and the necessary training was provided to the participants based on these instructions. At the beginning of the session, participants received additional explanations about the study’s objectives and the method of Q-sorting. After ensuring they understood the completion process correctly, they were asked to complete the Q-grid. Then, the participants were asked to sort the numerical values of the presented Q-statements based on their preferences within the score range of −5 to +5 on the Q-grid (Fig 1). In sorting the statements in the Q-grid, the statements are classified relative to each other in a normal distribution to provide a comprehensive picture of the participant’s subjectivities [44]. After Q-sorting, the researcher checked the completed grid by interacting with each participant.

### Step 4: Data analysis

Factor analysis of Q-sort statements was conducted using KADE (software, version 1.2.1), a specialized software for data analysis in Q-methodology studies [45]. Centroid factor analysis and Varimax rotation were performed to extract factors. Centroid factor analysis is a method specific to Q-methodology studies that allows researchers to examine each data set from different subjectivities before rotating it and identifying the best dominant subjectivities [33]. In this process, individuals are categorized instead of variables. Factors were retained according to the framework of Watts and Stenner [46]. In a 2‑hour session attended by three researchers (MKM, AN, and RH), the fulfilment of three criteria including 1) statistical criteria (eigenvalues of >1.00, The explained variance is a minimum of 35%, at least two Q-sorts in each factor), 2) qualitative criteria (the data obtained from the interviews should confirm the factors) and 3) methodological criteria (the factors should be differentiated, coherent, and recognizable) [33,46]. Furthermore, using the KADE software and selecting Auto-Flag, participants whose responses to the statements were not statistically significant at the p < 0.05 level compared to others in each factor category were excluded.

### Step 5: Factor interpretation

Factor arrays (weighted mean of Q-sorts) were calculated to interpret the results. The research team also combined quantitative (from the Q-sort rankings) and qualitative data (from the semi-structured interviews conducted during the Q-Set Development stage) to enrich the factorial interpretation of dominant subjectivities related to identifying factors influencing the selection of composite role models. Then, by examining the Q-statements that received the highest and lowest ranks in each factor, as well as the statements that were significantly distinguished from the others, a specific name and description were assigned to each factor using a subjectivity approach.

### Ethical consideration

The ethics code of this study was obtained (IR.TUMS.MEDICINE.REC.1402.245) from the Research Ethics Committee of the Faculty of Medicine, Tehran University of Medical Sciences. This study was conducted in accordance with the principles of the Declaration of Helsinki.Before participation, informed consent was obtained from all participants via a written form they reviewed and signed. This form outlined the study’s purpose, procedures, potential risks and benefits, and their rights, including the right to withdraw at any time without penalty. Participants were assured of the confidentiality of their information and that each interview would be coded to maintain anonymity. Participants were allowed to withdraw from the study at any time.

## Results

In the Q-sorting step, 31 medical students from the clerkship (n = 16) and internship (n = 15) phases of the undergraduate medical education at TUMS participated, comprising 14 males and 17 females with an average age of 23.8. In this study, following the mentioned criteria for evaluating the obtained factors, four main subjectivities were identified, which explained approximately 50% of the total variance [46]. The identified subjectivities are shown in Table 1. In the following, the description related to each subjectivity is presented. The information in parentheses includes: (the statement number, the score of that statement, and D for differentiating results).

**Table 1:** Q-sorts loading the four subjectivities.

Subjectivity	Loading Q-sorts	Number of Q-sorts	Eigenvalues	% variance
1	7, 10, 25, 24, 3, 19, 27, 15, 2, 20, 4, 17, 21	13	10.62	34
2	18, 8, 31, 12	4	1.84	6
3	16, 28, 14, 5, 1, 11	6	1.73	6
4	26, 23, 29, 30, 6	5	1.18	4

### The first subjectivity relies on professional and skillful role models

The first subjectivity explained about 34% of the variance, with 13 students included in this category. Table 2 presents the important statements in the first subjectivity category. After examining the statements, it was found that students with this subjectivity in choosing role models are looking for teachers who are committed to professional behaviors in their interactions with students and possess the competencies necessary to carry out their educational responsibilities. Participants with this subjectivity attach the most importance to the professional behaviors of the teacher and his or her commitment and responsibility (S 1 and 9: +5), and they pay special attention to the teachers’ abilities as role models. In this context, role models should demonstrate effective teaching skills (S 25: +4, D*) and allocate sufficient time to teaching students (S 25: +4, D*). Medical students with this subjectivity expect that role models will attend to students’ diverse needs (S 26: +3, D*) and, by providing feedback and encouraging reflection, create opportunities for their progress (S 23: +2, D*). In this context, medical students are not seeking alignment and similarity between themselves and the role model. Also, they do not look for cultural, religious, and political (S 42: −3) and personal development alignment with role models (S 41: −4). Moreover, having personal interests similar to those of students is not necessary for role models (S 45: −4, D*), and having a healthy lifestyle (S 3: −1, D*) is not a crucial statement for medical students in this context. The income level/economic status and successful family life (S 40 and 38: −5) were the least important among participants with this subjectivity. These results suggest that when selecting composite role models, the teachers’ personal lives are the least important factor for this group of medical students.

**Table 2:** Results regarding the first subjectivity rely on professional and skillful role models (N = 13, Variance explained = 34%).

No. Statement	Statements	Factor array	Distinguishing Statement(p < 0.05)
	**Highest-ranking statements**		
1	Professional behaviors	+5	
9	Commitment and responsibility	+5	
	**Highest-ranking statements compared to other subjectivities**		
27	Time spent teaching students	+4	D
25	Effective teaching skills	+4	D
26	Attention to student needs	+3	D
23	Paying attention to reflection and feedback	+2	D
	**Lowest-ranking statements compared to other subjectivities**		
3	Healthy lifestyle	−1	D
42	Cultural/religious/political alignment	−3	
41	Personal development alignment	−4	
45	Shared student interests	−4	D
	**Lowest-ranking statements**		
40	Income level and economic status	−5	
38	Successful family life	−5	

Full statement wording is provided in S1 Appendix.

### The second subjectivity relies on popular and famous role models

This subjectivity explained about 6% of the variance and included four students in this group (Table 3). After examining the statements, it was noted that medical students with this subjectivity are seeking role models who have achieved great fame at both micro and macro levels, such as those providing clinical services to patients or engaging in international communication, due to their extensive experience. The most important statements in this subjectivity are the role models’ ability to establish international communication and the popularity of the role model among patients (S 31 and 54: +5, D*). Participants with this subjectivity seek role models with up-to-date professional knowledge (S 21: +4) and can make effective environmental changes (S 35: +4). In this subjectivity, role models should be acceptable to students (S 52: +3) and have a high social status and dignity (S 37: +3, D*).

**Table 3:** Results regarding the second subjectivity rely on popular and famous role models (N = 4, Variance explained = 6%).

No. Statement	Statements	Factor array	Distinguishing statement(p < 0.05)
	**Highest-ranking statements**		
31	International communication and global acceptance	+5	D
54	Popularity among patients	+5	D
	**Highest-ranking statements compared to other subjectivities**		
35	Societal influence	+4	
21	Up-to-date professional knowledge	+4	
37	Social status and dignity	+3	D
52	Having acceptability in terms of the effectiveness of the on students and others	+3	
39	Matching the socio-economic situation of the country	+1	D
	**Lowest-ranking statements compared to other subjectivities**		
5	Venerable personality	−1	D
8	Humility and modesty	−2	D
16	Calmness and self-control	−3	D
	**Lowest-ranking statements**		
36	Having voluntary participation in academic activities	−5	
38	Successful family life	−5	

Full statement wording is provided in S1 Appendix.

Furthermore, compatibility with the socio-economic situation of the country (S 39: +1, D*) was considered one of the crucial statements in this subjectivity category. Medical students often overlook the importance of their role models’ personality traits. Having a distinguished, eminent, and venerable personality (S 5: −1, D*), as well as humility and modesty (S 8: −2, D*), and maintaining calmness and self-control (S 16: −3, D*) are not particularly important for these medical students. In this regard, the statements of the voluntary participation of the role model in academic activities (S 36: −5) and the successful family life of the role model (S 38: −5) have minor importance in this subjectivity.

### The third subjectivity relies on venerable and value-oriented role models

This subjectivity explained about 6% of the variance, and 6 participants were classified in this subjectivity category (Table 4). Medical students with this subjectivity seek teachers who define their behaviors within a specific, value-oriented framework and adhere to moral principles when choosing role models. Paying attention to the role models in observing and adhering to ethics and moral issues (S 4: +5, D*) and their distinguished, eminent, and venerable personalities (S 5: +5) are the most important statements in this category. In this subjectivity, the role models should have a value system aligned with the students in their profession (S 43: +4) and treat others politely (S 2: +4). Examining the statements in this subjectivity determined that the ruling values in the environment of encountering role models (S 49: −4, D*) are not considered important for these students. Also, medical students with this subjectivity, in choosing role models, consider role models’ abilities such as time management skills (S 6: −2, D*), flexibility towards issues (S 14: −1, D*), influence in the environment (S 35: −1), and the acceptability of role models among colleagues (S 53: −2, D*) are not considered. In this regard, the role models’ ability to establish international communication, foster global acceptance, and be familiar with and use modern educational technologies (S 31 and 24: −5, D*) were the least important to students with this subjectivity.

**Table 4:** Results regarding the third subjectivity rely on venerable and value-oriented role models (N = 6, Variance explained = 6%).

No. Statement	Statements	Factor array	Distinguishing statement(p < 0.05)
	**Highest-ranking statements**		
4	Adhering to moral principles	+5	D
5	Venerable personality	+5	
	**Highest-ranking statements compared to other subjectivities**		
2	Polite interactions	+4	
43	Professional value alignment	+4	
	**Lowest-ranking statements compared to other subjectivities**		
14	Flexibility toward issues	−1	D
35	Societal influence	−1	
7	Multidimensional skill enhancement	−2	
53	Acceptability among colleagues	−2	D
6	Time management skills	−2	D
49	Aligning with the perceived values of the environment	−4	D
	**Lowest-ranking statements**		
31	International communication and global acceptance	−5	D
24	Familiarity with and using modern educational technologies	−5	D

Full statement wording is provided in S1 Appendix.

### The fourth subjectivity relies on social and responsive role models

This subjectivity explained approximately 4% of the variance; five participants fell into this subjectivity category (Table 5). In this category of subjectivity, students emphasize the role models’ social activities and positive impact on society when choosing a role model. According to the statements that got the highest rank in this subjectivity, the role models should have a distinguished, eminent, and venerable personality and have commitment and responsibility towards the assigned tasks in the community of practice, society, etc. (S 5 and 9: +5). Medical students with this subjectivity seek role models who have established an effective relationship with others (S 30: +4, D*) and strive to engage in altruistic activities for the benefit of others and society (S 34: +3, D*). On the other hand, by examining the statements with the less importance, we find out the type and conditions of the encounter between the students and the role models, such as the number and variety of opportunities to meet (S 48: −3, D*), the students’ experience of educational-research collaboration with the role models (S 50: −3, D*) and role models’ field of work and expertise (S 44: −3, D*), has little importance in choosing them as role models. In this regard, role models’ income level and economic status (S 40 and 46: −5) formed the less important statements in this subjectivity.

**Table 5:** Results regarding the third subjectivity rely on social and responsive role models (N = 5, Variance explained = 4%).

No. Statement	Statements	Factor array	Distinguishing statement(p < 0.05)
	**Highest-ranking statements**		
5	Venerable personality	+5	
9	Commitment and responsibility	+5	
	**Highest-ranking statements compared to other subjectivities**		
30	Effective communication	+4	D
34	Altruistic activities	+3	D
	**Lowest-ranking statements compared to other subjectivities**		
48	Creating multiple opportunities and a variety of opportunities for students to be exposed	−3	D
50	Research/educational collaboration	−3	D
44	Aligning the field and expertise with the student’s favorite field	−3	D
	**Lowest-ranking statements**		
40	Income level and economic status	−5	
46	Student’s educational level	−5	

Full statement wording is provided in S1 Appendix.

## Discussion

Medical students construct composite role models by selectively integrating diverse factors, including individual dimensions, professional competencies, ethical values, culture, social dimensions, and institutional dimensions, from multiple sources.[18,47,48]. In this research, we aimed to elucidate the dominant subjectivities of medical students when selecting composite role models. We employed Q-methodology to investigate how clinical-phase medical students select and create composite role models, providing them with a Q-set of statements that represent diverse factors influencing their selection of composite role models [30]. Through their prioritization, we identified four distinct subjectivities for role model selection among students of the clinical phase of the undergraduate medical curriculum, which describe the shared subjectivities of students about factors influencing the choice of composite role models. These four subjectivities included 1) relying on professional and skillful role models, 2) relying on popular and famous role models, 3) relying on venerable and value-oriented role models, and 4) relying on social and responsive role models. These findings extend prior research, which often emphasized emulating a single role model and singular role model attributes [12,49], by highlighting students’ multifaceted approach to role model selection and student-driven construction of role models.

While some of the subjectivities identified in this study may be comprehensible to a global audience, others, such as ‘relies on venerable and value-oriented role models,’ are more deeply shaped by Iranian cultural and contextual factors, reflecting the context-dependent nature of role modeling where culture, ethical values, and societal norms influence the selection process [22,49,50]. Moreover, it is essential to emphasize that generalizability in Q-methodology differs fundamentally from that in conventional quantitative research. Since Q-methodology is not designed to estimate global population frequencies, the subjectivities identified here should not be interpreted as representing prevalence rates among all medical students. Instead, they reflect theoretically meaningful patterns in how students may construct composite role models within a complex sociocultural framework. such findings are transferable at the level of theory: they can inform hypotheses, generate mid-range conceptual models, and be compared across diverse contexts. Replication in other cultural and institutional settings, or follow-up with mixed methods, can help test whether these subjectivities recur and thus broaden their applicability [51]. In this sense, while the statistical generalizability of our findings is limited, their theoretical and interpretive value extends beyond the Iranian context. It may contribute to international discussions on how medical students conceptualize role models.

The findings of this study demonstrated that, for some students, the teacher’s professional behavior and instructional skills have a significant impact on how they select the role model to emulate. Students with dominant subjectivities that “rely on professional and skillful role models” tend to choose role models with high accountability and dedication. In previous studies, professional behaviors have been identified as one of the essential components in role modeling, and possessing characteristics such as professionalism and responsibility has been found to increase the likelihood of students being chosen as positive role models [52,53]. In line with previous studies, students in this subjectivity paid great attention to the role model teachers’ competencies [48]. The qualities of a positive role model include a teacher’s ability to educate and allocate sufficient time for instruction [48,54]. A role model should also be dedicated to their responsibilities, be punctual, and consider the students’ needs [52]. While it is true that a role model’s similarity to a student can be a facilitating aspect of role modeling [55]Students in this study were unconcerned about their alignment and similarity. Given that this subjectivity explained 34% of the variance in the selection of composite role models, it may initially reflect the academic culture at Tehran University of Medical Sciences, where professional behaviors and teaching competencies are emphasized [56]. However, students with this subjectivity tended to place little importance on similarity and alignment in areas such as shared values when choosing role models, suggesting it is not solely tied to the local context. Instead, considering that studies in other countries have also identified professional behaviors and teaching competencies as key features of positive role models [48,53], this emphasis may reflect broader, cross-cultural patterns in constructing composite role models, despite its prominence in the Iranian setting.

Many studies have highlighted the importance of personality characteristics in a role model and have identified factors such as humility, calmness, and self-control as key considerations in selecting a role model. This is despite the fact that, according to our findings, the students were categorized as “relying on popular and famous role models,” and the teacher’s personality traits were not a factor in their choice of role models. These students mostly look for teachers who have gained a great deal of fame and popularity among patients and at the national and international levels due to their extensive knowledge and vast connections, and are highly respected by students. Individuals are chosen as role models due to their high popularity, status, and social prestige, and they influence others. This preference may reflect implicit biases toward visible and prestigious figures, potentially overshadowing other qualities like ethical values or social responsiveness [2]. The results of Yazigi et al.’s study, which showed that role models’ professional image and credibility are considered less important [47], do not apply to students with the above subjectivities.

When selecting a role model, students who have a subjectivity of “relying on venerable and value-oriented role models” in this study tended to choose teachers who adhere to ethical principles rather than considering a role model’s acceptance and worldwide credibility as well as their ability to establish international communication. This group considers personality attributes to be crucial in selecting a role model. In this regard, along with previous studies, a distinguished, eminent, and venerable personality, as well as other personality traits, were considered essential in choosing the composite role models [47]. Additionally, aligning the role model’s behavior with the values of the medical profession and its significance in the student’s mind was crucial in selecting the role model [13]. As in Horsburgh et al.’s study, medical students with this subjectivity attached great importance to the alignment of the role models’ behaviors with the values of the medical profession [13]. This subjectivity, more context-dependent than others, is deeply influenced by Iranian cultural values, ethical principles, and the reverence for venerable personalities, shaping the selection of composite role models in a way that prioritizes moral alignment over visibility or power.

When considering a subjectivity of “relying on social and responsive role models,” educators who are interested in social issues and aim to have a beneficial impact on society are chosen as role models. With this subjectivity, medical students select a role model based on characteristics such as good interpersonal communication, commitment and accountability to the community, and altruism toward others, all of which have been suggested in earlier research, including the systematic study by Leeuw et al. [21].

To summarize, our research suggests that several factors play a significant role in selecting a composite role model for students with diverse subjectivities. For instance, the professional ability and competence of the teacher are crucial for students who have the dominant subjectivity of “relying on professional and skillful role models,” but it is not as significant for those who have the subjectivity of “relying on venerable and value-oriented role models,” or the qualities For individuals who have the subjectivity of “relying on venerable and value-oriented role models,” the role model’s personality is the most crucial component; however, students who fall into the category of “relying on popular and famous role models” and disregard it are thought to find it unimportant.

### Strengths and limitations

The use of Q-methodology in this study led to the identification of the subjectivities of medical students regarding the selection of role models. By conducting semi-structured interviews with medical students during the clinical phase and reviewing the literature, we gained an understanding of and explored the various influential factors in choosing composite role models. Although we aimed for maximum diversity in our sampling, potential biases may still exist that could affect the applicability of the findings to all medical students in the clinical phase. Deciding on the number of statements and choosing their degree of partiality and generalization for Q-sorting so that we could both ensure the validity of the statistical tests and avoid the excessive difficulty of completing the Q-grids created challenges. On the other hand, due to the students’ unfamiliarity with this study method and the time-consuming process of sorting the statements in the Q-grid, it wasn’t easy to invite students to participate in the study. However, we attempted to resolve the issue by designing suitable instructions for filling in the grids and arranging face-to-face meetings at the desired time and place for the participants.

Additionally, it should be noted that Q-methodology does not provide statistical generalizability to all medical students; rather, it identifies theoretically meaningful patterns of subjectivities. The findings are inherently shaped by the Iranian cultural and institutional context, and their applicability to other settings should be considered cautiously, ideally through replication or mixed-method follow-ups.

## Conclusion

It can be challenging for medical teachers to consider the factors in selecting role models that take into account students’ subjectivities. In the current study, we have recognized four subjectivities that explore how medical students select composite role models. We have employed cognitive construction approaches to describe the diversity in medical students’ subjectivities regarding the selection of role models. Teachers and medical schools can utilize these subjectivities to reflect on the factors involved in role modeling and relate them to the diverse perceptions of medical students. Various stakeholders can use the identified subjectivities to strengthen the factors involved in role modeling and improve the teaching-learning process. Also, the results of this study can be used to introduce and appreciate influential teachers in medical schools. Teachers and medical schools can apply these insights by developing faculty programs that acknowledge diverse role-modeling styles, recognizing students’ value for professional competence, popularity, value orientation, and social responsiveness. Additionally, they can use structured student feedback to identify a broader range of role models beyond traditional faculty, while integrating these findings into curriculum design to expose students to complementary role models, supporting professional identity formation and diverse career aspirations for a more inclusive teaching environment.

Additionally, this study contributes theoretically by showing how role modeling in medical education develops through a multifaceted and complex process. The four identified subjectivities create a helpful framework that points out both universal patterns (such as valuing professional competence) and culturally specific orientations (such as Venerable and Value-Oriented Role Models). These findings expand current theory by demonstrating how students actively construct composite role models, rather than passively emulating singular figures. Theoretical generalizability of these patterns can inform future hypothesis‑driven studies and cross‑cultural comparisons, thereby refining models of role modeling in medical education.

## Supporting information

S1 Appendix The complete list of Q statements. (DOCX)

S1 Data Anonymized Q-sort scores dataset. This file contains the anonymized dataset with individual Q-sort scores assigned to each statement by all participants, ensuring transparency while maintaining participant confidentiality. Further details or clarifications may be obtained from the corresponding author upon reasonable request.
(XLSX)
