# Supplementary material for: Understanding diverse subjectivities of medical students in seeking composite role models: A Q-methodology study
Source: PLoS One. 2026 Feb 27;21(2):e0339766. doi: 10.1371/journal.pone.0339766 (PMC12948135; doi:10.1371/journal.pone.0339766)
Supplement: S1 Appendix — (DOCX) [file pone.0339766.s001.docx]

S 1:Appendix. The complete list of Q statements.

| No. Statement | Statements |
| --- | --- |
| 1 | Having professional behaviors |
| 2 | Having polite interactions with others |
| 3 | Having a healthy lifestyle |
| 4 | Adhering to moral principles |
| 5 | Having a distinguished, eminent, and venerable personality |
| 6 | Having time management skills |
| 7 | Being multidimensionality and multifaceted Skill Enhancement |
| 8 | Having humility and modesty |
| 9 | Having commitment and responsibility |
| 10 | Having self-confidence |
| 11 | Being diligent and hardworking |
| 12 | Physical attributes and appearance |
| 13 | Being youthful |
| 14 | Having flexibility toward issues |
| 15 | being inspiring |
| 16 | Maintaining calmness and self-control |
| 17 | Being supportive and reliable |
| 18 | Having the cognitive ability and problem-solving |
| 19 | Having maturity and plenty of job experience |
| 20 | Mastering technical and specialized skills |
| 21 | Having up-to-date professional knowledge |
| 22 | Having a good CV |
| 23 | Paying attention to reflection processes and providing feedback |
| 24 | Familiarity with and using modern educational technologies |
| 25 | Having effective teaching skills |
| 26 | Paying attention to the needs of students |
| 27 | Spending time to teach students |
| 28 | Enticing and motivating students to engage in the learning process |
| 29 | Being able to assist students in reaching their aptitudes |
| 30 | Communicating effectively with others |
| 31 | Having international communication and global acceptance |
| 32 | Having the ability to collaborate in interdisciplinary teams with different professions |
| 33 | Giving advice and motivating students to lead moral lives |
| 34 | Having altruistic activities for others and society |
| 35 | Changing and influencing society |
| 36 | Having voluntary participation in academic activities |
| 37 | Having social status and dignity in society |
| 38 | Having a successful family life |
| 39 | Matching with the socio-economic situation of the country |
| 40 | Income level and economic status |
| 41 | Aligning with the personal development of students |
| 42 | Aligning with the cultural, religious, and political orientations of students |
| 43 | Aligning with student’s professional value system |
| 44 | Aligning the field and expertise with the student’s favorite field |
| 45 | Aligning the individual interests of students |
| 46 | The student’s educational level when exposure |
| 47 | Physical conditions of the environment when exposure |
| 48 | Creating multiple opportunities and a variety of opportunities for students to be exposed |
| 49 | Aligning with the perceived values of the environment |
| 50 | Collaborating in research/educational activities with students |
| 51 | Having academic acceptability among students |
| 52 | Having acceptability in terms of the effectiveness of the on students and others |
| 53 | Having acceptability among other colleagues in interpersonal and team interactions |
| 54 | Having popularity and reputation among patients |
| 55 | Introducing and appreciating the distinguished performance of role models |
